# Depression and the Use of Selective Serotonin Reuptake Inhibitors in Patients with Acute Intracerebral Hemorrhage

**DOI:** 10.7759/cureus.5975

**Published:** 2019-10-23

**Authors:** Maryam J Syed, Salman Farooq, Sarwar Siddiqui, Safia Awan, Mohammad Wasay

**Affiliations:** 1 Neurology, Aga Khan University Hospital, Karachi, PAK; 2 Internal Medicine, Aga Khan University Hospital, Karachi, PAK

**Keywords:** stroke, post-stroke depression, depression, selective serotonin reuptake inhibitors (ssri), intracerebral hemorrhage

## Abstract

Introduction

Depression is a common psychiatric complication associated with stroke. However, while most studies focus on post-stroke depression (PSD) subsequent to ischemic strokes, fewer studies have specifically explored depressive symptoms and the use of selective serotonin reuptake inhibitors (SSRIs) in patients with acute intracerebral hemorrhage (ICH). The aim of our study was to identify the incidence and factors associated with depression in ICH patients and the use of SSRIs as therapy by physicians at a tertiary care hospital in Karachi, Pakistan.

Materials and methods

A retrospective chart review was conducted to identify patients with ICH through the International Classification of Diseases, Ninth Revision (ICD-9) coding system electronic medical records of Aga Khan University Hospital, Karachi, Pakistan. Patient records spanning a period of five years at the hospital were identified and analyzed by neurology residents. Patients' clinical, laboratory, radiological, and pharmacological data were recorded and analyzed using a structured proforma. Patients with a past history of depression or those who were taking SSRIs at the time of admission were excluded from the analysis. Depression was defined as the presence of five or more symptoms according to the diagnostic criteria of the Diagnostic and Statistical Manual of Mental Disorders, Fourth Edition (DSM-IV).

Results

Out of the 458 patients we analyzed, 258 (56%) were men and 200 (44%) were women. The mean age was 59 years. Median National Institutes of Health Stroke Scale (NIHSS) score on admission was 13 (range: 0-42), and the median modified Rankin Scale (mRS) score was 4 (range: 0-6). On neuroimaging, sites of hemorrhage in patients were found to include the basal ganglia/thalamus in 279 (61%) patients, cerebral cortex in 105 (23%), cerebellum in 25 (5%), brain stem in 17 (4%), ventricles in 17 (4%), and multiple sites in eight (2%). We found that 48 (10%) patients had a ventricular extension, and 130 (28%) had midline shift, hydrocephalus, or both. Overall, 103 (22%) patients met the DSM-IV diagnostic criteria for depression. The most common depressive symptoms included tearfulness (67%), sadness (55%), and loss of interest or pleasure in life activities (53%). None of the patients reported suicidal ideation. Only seven patients (2%) were seen by a psychiatrist. The presence of depression was not significantly associated with hemorrhage sites [prabability value (p): 0.55] or the extent of disability (p: 0.09). Among the 103 depressed patients, only 25 (24%) received SSRIs during the hospital stay. A total of 57 (12%) received SSRIs during the hospital stay, of which only 25 had met the DSM-IV diagnostic criteria for depression. The mean duration between the diagnosis of ICH and the start of SSRIs was five days (range 3-25 days). None of the patients received any psychotherapeutic help for depression. At the time of discharge, only 13 (13%) of the 103 patients diagnosed with depression were discharged on SSRIs, while 23 that had not met the DSM-IV diagnostic criteria were discharged on SSRIs.

Conclusion

The present study demonstrates that depression is not uncommon in acute ICH patients, and it is both underdiagnosed and inadequately treated. Physicians should be trained to accurately identify and effectively treat depressive symptoms in ICH patients. Clear guidelines should be developed to aid the diagnosis and treatment of post-ICH depression in hospital settings.

## Introduction

The incidence of depression has been reported in one-third of all stroke patients [1,2]. Although most of the previous reports on post-stroke depression (PSD) are related to ischemic strokes, recent studies that focused on hemorrhagic strokes have shown that depression is not uncommon in intracerebral hemorrhagic (ICH) patients [3-6]. One study has found the prevalence of depression to be 63.3% (at one week) and 44.4% (at one month) in cases of acute ICH [7]. Very few studies have documented the incidence, diagnosis, and management of PSD in developing countries. The pathophysiology of depression in the very early phase of stroke is not well understood [1,8]. Most of the guidelines related to the treatment of stroke and ICH do not address the optimal ways to identify and treat depression in these patients. One major reason for the unavailability of clear guidelines may be the lack of evidence. A Cochrane review has concluded that there is limited evidence regarding the effectiveness of pharmaceutical interventions for PDS, while psychological interventions may indeed reduce the risk of its occurrence [9].

Selective serotonin reuptake inhibitors (SSRIs) have been reported to have a small but significant positive effect in treating PSD [1,9]. The cerebrovascular effects of SSRIs have been a topic of discussion in recent years. Using SSRIs, especially along with other anticoagulants, has been shown to increase the risk of ICH in the first month of use [10]. SSRIs may affect platelet aggregation and thus increase the risk of bleeding. However, other studies have shown this risk to be insignificant [11]. The safety of SSRIs for depressive symptoms in patients with ICH is yet to be evaluated in well-designed, prospective trials. There is a paucity of information about how commonly SSRIs are used in patients with acute stroke or ICH, and how frequently this use is based on established diagnostic criteria. The objective of our study was to identify the frequency of depression and the use of SSRIs in patients with ICH at a tertiary care center in Pakistan.

## Materials and methods

This retrospective study was conducted from August 2018 to October 2018 at Aga Khan University Hospital, Karachi, and was approved by the university’s Institutional Review Board. Data were electronically retrieved and patients with ICH and depressive symptoms were identified through the International Classification of Diseases, Ninth Revision (ICD-9) coding system of the hospital's electronic medical records. The primary objective was to record and document the frequency of depressive symptoms in ICH patients and to examine how often SSRIs were utilized as therapy, whereas the secondary objective was to document associated factors including age, gender, site of hemorrhage on neuroimaging, comorbidities, and patient disability based on National Institutes of Health Stroke Scale (NIHSS) and modified Rankin Scale (mRS) scores. A retrospective chart review of medical records spanning a period of five years was conducted by neurology residents. Patients' clinical, laboratory, radiological, and pharmacological data were recorded and analyzed using a structured proforma. Depression was defined as the presence of five or more symptoms as per the Diagnostic and Statistical Manual of Mental Disorders, Fourth Edition (DSM-IV) criteria, irrespective of their duration. Patients were excluded if they had a prior history of depression or if they were taking SSRIs at admission. Statistical Package for the Social Sciences (SPSS) version 24 (IBM, Armonk, New York) was used to enter and analyze data. Frequencies were calculated using descriptive statistics. Significant associations between depression and site of hemorrhages were analyzed using Pearson chi-square and Fisher’s exact tests. A probability value (p) of less than 0.05 was considered statistically significant in all cases. The absolute and relative (percentage) frequency and types of depressive symptoms were reported.

## Results

Out of the 467 patients with ICH that we identified, nine (2%) had a past history of depression and were taking SSRIs at admission. They were excluded from the analysis, leaving us a total of 458 patients for the study: 258 (56%) men and 200 (44%) women. The mean age of the patients that we selected was 59 years (range: 12-99 years). Here we summarize the frequencies and percentages of comorbidities in ICH patients in our study (Table 1).

**Table 1:** Comorbidities of ICH patients included in the study ICH: intracerebral hemorrhage

ICH-associated risk factors	Number of patients	Frequency, %
Hypertension	355	77.5
Diabetes mellitus	115	25
Cigarette smoking	45	10

The sites of hemorrhage in patients as seen on neuroimaging findings are tabulated below (Table 2).

**Table 2:** Frequency and sites of ICH on NCCT scan of the head ICH: intracerebral hemorrhage NCCT: non-contrast computed tomography

Sites of hemorrhage on NCCT	Number of patients	Frequency, %
Basal ganglia/thalamus	279	61
Brain stem	17	4
Ventricles	17	4
Multiple sites	8	2
Ventricular extension	48	10

The median NIHSS score on admission was 13 (range: 0-42), and the median mRS score was 4 (range: 0-6). Interestingly, 130 (28%) patients had also shown radiological signs of midline shift, hydrocephalus, or both. Overall, 103 (22%) met the DSM-IV diagnostic criteria for depression. The most common depressive symptoms included tearfulness (67%), sadness (55%), and loss of interest or pleasure in life activities (53%). None of the patients reported suicidal ideation. Only seven (2%) were seen by a psychiatrist. The presence of depression was not directly related to the site of hemorrhage (p: 0.55) or the extent of disability (p: 0.09). Among the 103 depressed patients, only 25 (24.27%) received SSRIs during their hospital stay. A total of 57 (12%) ICH patients received SSRIs during their hospital stay, out of which only 25 had met the DSM-IV diagnostic criteria for depression. The mean duration between the diagnosis of ICH and the start of SSRIs was five days (range: 3-25 days). None of the patients received any psychotherapeutic interventions for depression. At the time of discharge, only 13 of the 103 patients diagnosed with depression were discharged on SSRIs, while 23 patients that had not met the diagnostic criteria of depression were still discharged on SSRIs.

## Discussion

We believe these findings provide useful insights into the frequency and management of depressive symptoms in stroke patients in a developing country. We found a discrepancy between the diagnosis of depression and the use of SSRIs for treatment among these patients. Half of the patients receiving SSRIs had not met DSM IV criteria for depression, while 75% of the patients meeting the criteria did not receive the appropriate treatment.

Depression is reported to occur in one-third of the patients suffering from stroke and is also associated with poor functional outcomes and high mortality [1]. Hackett et al. have reported the pooled frequency estimate of PSD to be 31% in the first five years after stroke [2]. Most of the studies on PSD either focus solely on ischemic stroke or do not differentiate between ischemic and hemorrhagic strokes in their analysis [3]. There is a dearth of knowledge regarding the incidence, severity, and etiology of depression in hemorrhagic stroke survivors, especially in developing countries [12]. The frequency of depression in our study (22%) was comparable to a few of the larger studies. Christensen et al. reported that 20% of the patients in the FAST (Factor Seven for Acute Hemorrhagic Stroke) trial reported depressive symptoms at three months of the onset of ICH, whereas another study reported the prevalence of depression at one year after ICH to be 15% [3,5]. Similarly, in a median follow-up at nine years, Koivunen et al. documented the prevalence of depressive symptoms in ICH survivors to be 23.1% [6]. On the other hand, Francis et al. reported a prevalence of only 3.8% at three months and 4.9% at 12 months in ICH patients [4]. The exact frequency of depression subsequent to ICH is still unknown. The reported prevalence values often vary between studies due to the observed heterogeneity in their methodology and designs; this includes inconsistencies in the sample patients (either consisting of patients from inpatient department of tertiary care hospitals or those from out-patient rehabilitation centers), the differences in the time period chosen to assess depression, as well as the numerous types of scales used to diagnose depression, all with different cut-off scores.

To the best of our knowledge, post-ICH depression has not been comprehensively studied in Pakistan. A cross-sectional study on the functional and cognitive outcomes of stroke patients in Pakistan (in which hemorrhagic strokes accounted for 26%) demonstrated a quarter of the survivors to be depressed [12]. Furthermore, hemorrhagic stroke is reported to be more common in Pakistan (21-31%) compared to the Western world (10-15%) [13,14]. This indicates that the incidence of post-ICH depression may be even higher in South Asian countries and underscores the importance of accurate diagnoses and treatment of depression in ICH patients. ICH is usually associated with poor health-related quality of life (HRQOL) [15]. Early and accurate identification and appropriate treatment of post-ICH depression are of vital importance for decreasing morbidity and mortality associated with it [1,8].

The most common depressive symptoms included tearfulness (67%), sadness (55%), and loss of interest or pleasure in life activities (53%). Similar symptoms were observed in acute ICH patients in other studies [5,7]. Other important findings significantly associated with post-ICH depression that can aid in diagnosis include sleep disturbances, psychomotor agitation, and loss of appetite [7]. Additionally, Sallinen et al. showed that feelings of depression prior to the onset of ICH serve as a significant predictor of developing post-ICH depression in patients [16]. SSRIs are the pharmacological treatment of choice in PSD due to their additional benefits of improving neurogenesis and neuroplasticity, which help in physical and cognitive recovery in stroke patients while also having the least cholinergic and cardiovascular adverse effects [8,17-19]. It may be argued that due to a possible increased risk of bleeding complications [10], SSRIs should be withheld from ICH patients. However, this risk has been described to be considerably low in some studies as they discerned only one additional intracerebral bleeding episode per 10,000 persons treated with SSRIs for one year [11]. SSRIs may be safely prescribed to treat post-ICH depression to outweigh the detrimental effects of depression on functional outcome and mortality [1].

Significantly, 75% of the patients meeting the diagnostic criteria did not receive appropriate treatment in our study. PSD is often unrecognized and inappropriately treated [4,8]. Diagnosis of depression, especially in acute stroke patients, has proven to be challenging. Acute stroke patients often present with neurological deficits of aphasia and associated linguistic difficulties, somatic symptoms, anxiety, as well as difficulty in cognition. These complications can frequently mask depressive symptoms and make it difficult for physicians to ascertain the presence of depression. The patients in our study were mostly treated by neurologists who may have overlooked depressive symptoms while treating more obvious complications of ICH. Despite the high prevalence of psychiatric comorbidities in neurological settings, studies have demonstrated that the rate of inpatient referrals made by neurologists to psychiatrists is surprisingly low [20,21]. Additionally, patients in our study may have been underdiagnosed and undertreated due to a lack of awareness regarding the identification of depression and its treatment among physicians treating stroke patients. Compared to the Western world, there is a lack of understanding regarding depressive disorders in South Asian countries, not just in the general population but also among physicians. An Indian study about the perception of depression among medical providers demonstrated gaps in knowledge of the etiology of depressive disorders, with many physicians attributing it solely to psychosocial circumstances rather than underlying biological mechanisms [22]. The same study also reported potential misconceptions about antidepressants and their use for depression. Given that the majority of stroke patients in Pakistan are managed by their general practitioners after discharge [21], these findings may also be generalizable for Pakistani physicians due to the cultural similarities between the two neighboring countries.

Half of the patients who did receive SSRIs had not met DSM IV criteria for depression in our study. A probable explanation of the high use of SSRIs in patients may be the overlap of stroke symptoms with depression symptoms, which may lead to a false-positive diagnosis of depression among stroke patients. A major reason for all discrepancies observed in our study could be a lack of clear guidelines regarding the use of SSRIs among these patients. American Stroke Association has not released any guidelines regarding assessment, treatment, and prevention of depression in ICH patients [1,4]. Considering the above-mentioned challenges in diagnosis, we believe that developing a specific screening tool for the identification of stroke-specific depressive symptoms, such as sensory loss and cognitive deficits, may increase the sensitivity of depression diagnosis in stroke patients [8]. None of the patients in our study underwent psychotherapy. There is a critical shortage of inpatient rehabilitation services or organized long-term home support services for stroke patients in Pakistan, as well as a lack of expertise to identify and treat associated mental disorders [23]. Due to a prevalent joint family system, stroke patients often have multiple family members and attendants who cater to their needs [12,23]. This system can benefit greatly from ecosystem-focused therapy that relies on the family’s participation and support [24,25]. Additionally, caretakers should be educated about depressive symptoms and encouraged to consult psychologists or psychiatrists.

We did not find any correlation between the location of hemorrhage on neuroimaging studies and depressive symptoms (p: 0.55). These findings are in agreement with previous reports [5,7,26]. However, some studies have found a significant association between PSD and left- hemisphere lesions [27]. The presence of depression was not found to be directly related to the extent of disability in our study. Depression has been reported to be more common in the setting of stroke when compared to any other ailments with the same level of disability [1,8]. This prompts us to believe that there is more to post-ICH depression than just an initial psychological response to the trauma of stroke and disability. There are contradictory results across studies regarding the relationship between post-ICH depression and the extent of disability, with some showing no significant association [5,24], while others reporting a strong association between the extent of disability and the presence of post-ICH depression [1,3,6]. Using patient-reported outcome measures, Katzan et al. reported that the patient groups with higher disability and poor HRQOL had significantly higher scores for depression, compared to groups with a mild-to-moderate disability and mixed HRQOL [28]. More research is required to understand the underlying pathophysiology behind ICH and subsequent depression. Studies utilizing advanced imaging techniques such as diffusion tensor imaging (DTI) and functional magnetic resonance imaging (fMRI) may shed some light on the relationship between lesion location and microstructural abnormalities associated with both ICH and depression [26]. 

There are several limitations to our study, which employed a retrospective chart-review method. Firstly, cases with depression were included based on diagnostic codes of DSM IV criteria for depression, and this may have decreased the accuracy of the diagnosis since holding live, structured interviews based on the updated DSM V criteria is currently the gold standard of diagnosing depression. Additionally, the mean duration of time between the diagnosis of ICH and the administration of SSRIs was five days. DSM IV requires at least two weeks of persistent depressive symptoms to confirm the diagnosis. Secondly, a number of patients may have been missed out due to a lack of documentation, and this may have resulted in incomplete case ascertainment. Furthermore, it is possible that not all depressive symptoms are accounted for in ICD-9 codes, which may have led us to underestimate the frequency of depression in acute ICH patients. Thirdly, ours was not a longitudinal study, and we did not screen patients for depressive symptoms at different intervals in time to document any changes in the frequency of depression. Our study was not able to answer any questions about the cause of depression in acute ICH patients. Further research is needed to replicate the findings of our study.

## Conclusions

We believe our study provides valuable insights into the frequency of depression in acute ICH patients in a lower-middle-income country like Pakistan and highlights the pertinent issues regarding its accurate diagnosis and treatment. Depression was present in 22% of patients with ICH. During the hospital stay, 75% of patients with depression were not treated with SSRIs. More than half of the patients treated with SSRIs did not fulfill the criteria for depression. Our study highlights the need for providing better training to physicians to enable them to identify and treat depression in acute ICH patients in order to improve their quality of life and functional outcomes.
